# A Case of Barbiturate Poisoning From Pentobarbital in a Young Japanese Patient

**DOI:** 10.7759/cureus.36498

**Published:** 2023-03-21

**Authors:** Yuki Koizumi, Michiaki Higashitani, Sayato Fukui, Taisuke Kodama, Hiroshi Ito, Daiki Kobayashi

**Affiliations:** 1 General Internal Medicine, Tokyo Medical University Ibaraki Medical Center, Inashiki, JPN; 2 Cardiology, Tokyo Medical University Ibaraki Center, Ibaraki, JPN

**Keywords:** borderline personality disorder, concomitant prescriptions, trifecta of barbiturate intoxication, pentobarbital, overdose

## Abstract

Concomitant prescriptions of psychotropic drugs such as sleeping pills, antidepressants, and anti-anxiety medications are common. The relationship between the number of psychotropic drug prescriptions and the incidence of drug overdose has not been reported. However, efforts have been made to reduce the number of concomitant prescriptions hoping that fewer prescriptions of multiple drugs will lower the incidence of drug overdoses. Furthermore, among sleeping pills, prescriptions of barbiturates have been gradually decreasing due to the risk of severe side effects and addiction. This report features a case of an overdose of pentobarbital tablets that caused the classic medical triad (impaired consciousness, hypotension, and hypothermia) of barbiturate intoxication under the characteristics of borderline personality disorder.

## Introduction

Recently, many psychiatric patients have been taking combinations of sleeping pills, antidepressants, and antianxiety drugs, regardless of the efforts to reduce the number of drugs prescribed concurrently [1]. Of the most commonly prescribed sleeping pills, barbiturate use has decreased owing to the high risk of adverse effects or intoxication [2,3]. With the increasing global precedence of drug use, a patient was admitted to the hospital with barbiturate poisoning by an overdose of pentobarbital tablets and presented with impaired consciousness, hypotension, and hypothermia [4].

Psychiatric patients receive polypharmacy comprising sleeping pills, antidepressants, and antianxiety drugs. Based on national data in Japan, the proportion of patients prescribed one, two, three, four, five, or more than five antipsychotic drugs at once were 32%, 24%, 13%, 11%, 5%, and 15%, respectively [5]. As multiple drug prescriptions may be associated with the incidence of overdose or severe adverse events, the government of Japan has attempted to limit the number of multiple drug prescriptions. Recently, revisions of medical fees that reduced the fee for simultaneously prescribed drug prescriptions have been implemented in 2016 and 2018 [6,7]. However, studies about the association between multiple drug prescriptions and the incidence of overdose are very limited [8]. In addition, certain types of drugs, such as pentobarbital which is still in the market in Japan but discontinued in the U.S., had special attention due to severe side effects [9]. Therefore, even monotherapy with such drugs may still present a high risk of suicidal overdose, sometimes resulting in fatal attempts. Herein is a case of barbiturate poisoning in a patient with borderline personality disorder.

## Case presentation

A 21-year-old Japanese woman was admitted to the hospital with the chief complaint of loss of consciousness. One month before admission, she had suicidal ideation. The suicidal ideation gradually increased after two weeks. On the day of admission, she was found unconscious by family members and was transported to the hospital by ambulance. Family members also found 30 empty press-through-pack (PTP) sheets of pentobarbital (50 mg) and suspected a suicidal attempt by pentobarbital overdose. She had a history of anorexia nervosa, borderline personality disorder, major depression (self-interrupted one year before admission), and overdose (hospitalized four times for medical care and protection). At the time of hospitalization, she was not taking any of her medications approximately for one year even though she was prescribed pentobarbital, sertraline hydrochloride, and risperidone. She was a lifelong non-smoker and abstained from drinking.

The patient's vital signs on admission were as follows: initial Glasgow coma scale (GCS) was E1V1M1 (level of consciousness), blood pressure of 82/48 mmHg, heart rate of 66 beats per minute, respiratory rate of 12 breaths per minute, a body temperature of 34.5°C (94.1°F), oxygen saturation (SpO2) of 100% (room air). She had glossoptosis and both her pupil diameters measured 2 mm. A laceration was found on her left wrist. Results from blood tests are shown in Table 1 and Table 2.

**Table 1:** The results of complete blood count and atrial gas analysis All results were obtained at the time of admission.

Complete blood count		Reference range (unit)
White blood cell	6,200	3,100-8,400 (/μL)
Hemoglobin	9.1	12-16 (g/dl)
Hematocrit	30.8	34-45 (%)
Mean corpuscular volume (MCV)	78	80-98 (fl)
Mean corpuscular hemoglobin (MCH)	23	25-34 (Pg)
Mean corpuscular hemoglobin concentration (MCHC)	29.5	31-36.9 (g/dl)
Platelet	269	150-450 (10^3/μL)
Atrial blood gas analysis		
Potential of hydrogen (pH)	7.321	7.35-7.45
Partial pressure of carbon dioxide (PCO2)	46.6	35-45 (mmHg)
Partial pressure of oxygen (PO2)	103	80-100 (mmHg)
Bicarbonate (HCO3)	23.5	23-28 (mmol/L)
Carboxyhemoglobin (COHb)	0.3	0.5-0.8 (%)
Methemoglobin (MetHb)	0.3	0.04-2 (%)
Lactate	7.4	3.3-14.9 (mg/dl)

**Table 2:** Results of chemistry All results were obtained at the time of admission.

Chemistry		Reference range (unit)
Total protein	6.4	6.5-8 (g/dl)
Albumin	3.4	3.9-4.9 (g/dl)
Total bilirubin	0.4	0.4-1.5 (mg/dl)
Aspartate aminotransferase (AST)	8	7-38 (U/L)
Alanine aminotransferase (ALT)	6	4-44 (U/L)
Lactate dehydrogenase (LDH)	154	120-220 (U/L)
Creatine phosphokinase (CPK)	157	30-200 (U/L)
Alkaline phosphatase (ALP)	37	50-350 (U/L)
Gamma-glutamyl transpeptidase (γ-GTP)	10	<30 (U/L)
Blood urea nitrogen (BUN)	4.5	8-20 (mg/dl)
Creatine (Cre)	0.55	<1 (mg/dl)
Sodium (Na)	144	137-147 (mEq/L)
Potassium (K)	3.5	3.5-5 (mEq/L)
Chlorine (Cl)	111	99-109 (mEq/L)
Calcium (Ca)	8.6	8.8-10.4 (mg/dl)
Magnesium (Mg)	2.2	1.8-2.6 (mg/dl)
Ionic phosphate (IP)	3.1	2.5-4.5 (mg/dl)
C-reactive protein (CRP	2.33	<0.3 (mg/dl)

The urinary test was positive only for pentobarbital indicating drug abuse, although the last prescription for barbiturates was approximately one year ago. Her serum pentobarbital concentration was 16.6 μg/mL (lethal concentration: more than 30 μg/mL) [10]. Electrocardiogram revealed normal sinus rhythm, normal axis deviation, a heart rate of 58 beats per minute, and no ST-T wave changes. Brain and whole-body computed tomography scans were unremarkable.

The patient was diagnosed with barbiturate poisoning secondary to a pentobarbital deliberate overdose. The treatment involved relieving the airway obstruction by inserting the nasopharyngeal airway to prevent glossoptosis, saline infusion and vasopressors for hypotension, and rewarming in an internal medicine ward. An infusion of sodium bicarbonate to promote urinary alkalinization was also administered, although the evidence of urinary alkalinization for improvement of mortality in patients with pentobarbital intoxication was limited [11]. She was also commenced on ampicillin/sulbactam for aspiration pneumonia. She did not receive gastric irrigation due to the unknown duration of overdose, lack of evidence of drug-induced respiratory suppression, and risk of additional aspiration. Her consciousness level improved three days following hospital admission, followed by temporary agitation, and she was discharged to a psychiatric unit.

## Discussion

The number of barbiturate prescriptions has decreased dramatically as non-benzodiazepine "Z" drugs, including the orexin receptor antagonist and melatonin agonist, have been associated with lower risks of adverse events or overdose and have therefore been increasingly prescribed instead of barbiturates [12]. The combination tablet of chlorpromazine and promethazine, now classified as a barbiturate sleeping pill, was discontinued in Japan in 2016. However, barbiturate sleeping pills are still prescribed, resulting in the resurgence of barbiturate intoxication [13]. Overdose by barbiturate intoxication may be suspected when patients present with impaired consciousness, hypotension, and hypothermia, as seen in this case. Table 3 shows symptoms in a patient with barbiturate intoxication [14]. 

**Table 3:** Symptoms in a patient with barbiturate intoxication

	Symptoms	
Non-fatal intoxication	Neurologic	Difficulty thinking, decreased level of consciousness, poor coordination, vertigo, nausea, muscle weakness, dilated or contracted pupils
	Cardiac	Bradycardia, rapid and weak pulse
	Systemic	Thirst, oliguria, decreased temperature,
Fatal intoxication		Coma, hypotension, respiratory depression, pulmonary edema

The incidence of overdose may be associated with patient characteristics. This patient was diagnosed with borderline personality disorder complicated by comorbid psychiatric disorders and had previously reported suicidal ideations [15]. This patient was diagnosed with major depression under the characteristics of borderline personality disorder and treated with pentobarbital, selective serotonin reuptake inhibitors (SSRI), and antipsychotics. The patient had made four suicidal attempts with suicidal ideations. About 10% of patients with borderline personality disorder commit suicide, and a further 80% to 90% of patients have a history of suicide attempts [16]. Patients with borderline personality disorder have a fear of abandonment [17], which may trigger non-life-threatening suicide attempts, such as overdose and wrist cutting [18]. However, these attempts may result in unexpected death. Each physician should pay attention to the number of antipsychotic prescriptions or wrist lacerations patients have to prevent undesirable patient outcomes.

Our patient had mild anemia. Although the anemia is a side effect of pentobarbital, possibly due to abnormality of folic acid metabolism, it is well-known and was presumed to be an iron deficiency anemia due to menstruation. Low mean corpuscular volume (MCV) suggested that the patient had iron deficiency anemia rather than anemia due to pentobarbital, which usually showed megaloblastic anemia. Moreover, a single overdose of pentobarbital does not cause anemia. In addition, this patient also had mild respiratory acidosis with an elevated partial pressure of carbon dioxide (PCO2). Special attention should be paid to prevent pulmonary arrest when a patient has respiratory acidosis under pentobarbital intoxication. 

Based on the findings from a national database in Japan, 92% of patients who had been prescribed a monotherapy of antipsychotic drugs did not owe any psychotherapy fee, which may suggest that they were prescribed these drugs by doctors other than psychiatrists [4]. Such patients may not be appropriately evaluated for their mental status or adherence to their prescription. However, it may sometimes be difficult to evaluate the patient’s adherence, even for psychiatrists, as seen in our patient who kept her pentobarbital tablets that had been prescribed by a psychiatrist one year before the overdose.

## Conclusions

The patient experienced poisoning due to an overdose of pentobarbital tablets under a monotherapy of pentobarbital that caused the classic medical triad of barbiturate intoxication. A patient who has such symptoms should be suspected for barbiturate intoxication even if the patient has a monotherapy of pentobarbital. Furthermore, special attention should be paid to patients with borderline personality disorder as they have a higher risk of suicidal behavior compared to the general population. In conclusion, developing an environment where each physician in non-psychiatric institutes can consult psychiatrists without barriers, and the removal of barriers to visiting psychiatric institutes are necessary to reduce the incidence of suicide.
